# Supplementary material for: Almond Consumption Improves Inflammatory Profiles Independent of Weight Change: A 6-Week Randomized Controlled Trial in Adults with Obesity
Source: Nutrients. 2026 Mar 9;18(5):875. doi: 10.3390/nu18050875 (PMC12986923; doi:10.3390/nu18050875)
Supplement: Supplementary file 1 [file nutrients-18-00875-s001.zip › nutrients-4165236-supplementary.pdf]

Table S1. Nutrient composition of almond and cookie study snacks in the 6-week randomized controlled trial in adults with obesity

	Almond[§]	Cookie[¶]
**Weight, g**	57	65
**Energy, kcal**	322*	325
**Carbohydrate, g**	12	43
**Dietary fiber, g**	6	1
**Protein, g**	12	3
**Total fat, g**	30	16
**Total SFA, g**	2	5
**Total MUFA, g**	19	4
**Total PUFA, g**	7	6
**Vitamin E (alpha-tocopherol), mg**	11	1
**Calcium, mg**	120	14
**Magnesium, mg**	160	27
**Iron, mg**	2	4
**Potassium, mg**	434	113
**Sodium, mg**	1	205
**Zinc, mg**	2	0

[§] Nutrient composition of natural, whole, unsalted, and dry roasted almonds provided by the Almond Board of California.
[¶] Nutrient composition of Chips Ahoy chocolate chip cookies obtained from food label and the USDA food composition database.
*Metabolizable energy calculated as the average of Atwater general factor estimates and measured energy estimates from Gebauer et al. 2016 [75].

Table S2. Self-reported dietary intake (energy-adjusted) from the ASA-24 dietary recalls for the almond and cookie groups at baseline and week 6 in adults with obesity.

	Baseline		Week 6		BL-adjusted Model P-Values	Linear Mixed Effect Model P-Values		
	**Almond**	**Cookie**	**Almond**	**Cookie**	**BL-adjusted Group**	**Group**	**Week**	**Group x Week**
**Carbohydrate (g)**	239.85 ± 9.34	235.59 ± 9.98	202.87 ± 9.51**	233.82 ± 10.31	0.086	0.257	0.005	0.009
**Total Fat (g)**	92.29 ± 3.79	85.41 ± 4.05	103.55 ± 3.86	90.75 ± 4.18	0.231	0.039	0.003	0.282
**Total MUFA (g)**	32.38 ± 1.48	28.56 ± 1.58	39.16 ± 1.51 *,**	28.58 ± 1.64	0.001	<0.001	0.004	0.004
**Oleic acid (g)**	30.51 ± 1.39	26.71 ± 1.48	37.07 ± 1.43 *,**	26.9 ± 1.54	0.003	<0.001	0.003	0.005
**Total PUFA (g) ǂ**	-0.13 ± 0.12	-0.12 ± 0.13	0.07 ± 0.13	0.2 ± 0.13	0.613	0.618	0.024	0.591
**Protein (g)**	82.73 ± 5.59	95.63 ± 5.96	94.68 ± 5.71	85.57 ± 6.18	0.061	0.783	0.832	0.012
**Total fiber (g) ǂ**	0.1 ± 0.12	-0.01 ± 0.13	0.01 ± 0.13	-0.1 ± 0.14	0.761	0.439	0.417	0.991
**Alpha-tocopherol (mg)**	11.5 ± 1.19	9.23 ± 1.26	16.45 ± 1.22	10.29 ± 1.32	0.004	0.002	0.005	0.067
**Calcium (mg)**	929.79 ± 78.81	985.31 ± 83.54	1044.67 ± 81.08	894.36 ± 87.15	0.474	0.581	0.876	0.175
**Magnesium (mg)**	319.36 ± 21.19	329.25 ± 22.53	365.3 ± 21.69	303.3 ± 23.41	0.084	0.302	0.577	0.041
**Phosphorus (mg)**	1357.08 ± 70.86	1479.21 ± 75.51	1540.84 ± 72.37	1311.89 ± 78.27	0.040	0.538	0.884	0.002
**Potassium (mg)**	2500.77 ± 123.5	2628.93 ± 131.01	2551.35 ± 126.85	2224.42 ± 136.52 ^^	0.176	0.477	0.121	0.043
**Zinc (mg) ǂ**	-0.04 ± 0.11	-0.07 ± 0.12	0.12 ± 0.12	-0.02 ± 0.12	0.571	0.429	0.373	0.620
**Refined grains (oz. eq.)**	5.36 ± 0.52	5.5 ± 0.55	5.53 ± 0.54	6.11 ± 0.58	0.495	0.528	0.447	0.659

Data are presented as model-adjusted least squares means ± SE. ǂ Indicates JN transformed variable means ± SE and P-values. *Mvt-adjusted pairwise P-value ≤ 0.05 for Almond vs. Cookie within the same timepoint, **Mvt-adjusted pairwise P-value ≤ 0.05 for BL vs. W6 within the same group, ^^Mvt-adjusted pairwise P-value ≤ 0.1 for BL vs. W6 within the same group

Repeated measures were analyzed using linear mixed-effects models (fixed effects: group, week, group x week; random intercept: participant) with energy intake included as a covariate. The Group, Week, and Group x Week columns report Type III tests of fixed effects from these models; pairwise comparisons used multivariate-t (mvt) adjustment. Baseline adjusted Group reports the group effect at Week 6 from a separate baseline-adjusted linear regression. Missing data were handled by maximum likelihood (mixed models) or multiple imputation (regression models).

Table S3. Immune and Inflammatory markers for the almond and cookie groups at baseline and week 6 in adults with obesity.

	Baseline		Week 6		BL-adjusted Model P-Values	Linear Mixed Effect Model P-Values		
	Almond	Cookie	Almond	Cookie	BL-adjusted Group	Group	Week	Group x Week
**Absolute basophils count (cells/uL)**	45 ± 3.01	40.9 ± 3.33	41.6 ± 3.17	41.16 ± 3.36	0.384	0.584	0.362	0.289
**Basophils %**	0.72 ± 0.04	0.65 ± 0.05	0.67 ± 0.04	0.66 ± 0.05	0.554	0.480	0.660	0.295
**Absolute eosinophils count(cells/uL)**	162.68 ± 16.34	149.03 ± 18.09	157.79 ± 16.87	142.72 ± 18.18	0.808	0.535	0.430	0.921
**Eosinophils %**	2.58 ± 0.22	2.33 ± 0.25	2.42 ± 0.24	2.25 ± 0.25	0.976	0.497	0.351	0.750
**Absolute monocytes count (cells/uL)**	439.24 ± 23.87	496.84 ± 26.43	427.35 ± 24.83	472.73 ± 26.59	0.631	0.124	0.122	0.600
**Monocytes %**	7.06 ± 0.35	8.08 ± 0.39	6.83 ± 0.38	7.83 ± 0.4	0.038	0.029	0.354	0.954
**Absolute neutrophils count (cells/uL)**	3711.45 ± 228.95	3713.29 ± 253.49	3813.15 ± 239.31	3624.96 ± 255.21	0.549	0.770	0.955	0.426
**Neutrophils %**	57.09 ± 1.48	57.01 ± 1.64	58.47 ± 1.57	58.13 ± 1.66	0.729	0.919	0.151	0.885
**Absolute lymphocytes count (cells/uL)**	2001.15 ± 90.75	1980.84 ± 100.47	1920.78 ± 95.05	1887.12 ± 101.19	0.802	0.830	0.074	0.891

**Lymphocytes %**	32.53 ± 1.33	31.93 ± 1.47	31.6 ± 1.41	31.11 ± 1.49	0.907	0.763	0.280	0.945
**White blood cells count (cells/uL)**	6.36 ± 0.29	6.38 ± 0.32	6.38 ± 0.3	6.17 ± 0.32	0.445	0.812	0.470	0.365
**TNF-alpha (pg/mL)**	31.2 ± 1.69*	25.17 ± 1.79	16.92 ± 1.72*,**	24.86 ± 1.8	<0.001	0.600	0.000	<0.001
**IFN-gamma (IU/mL)**	2.32 ± 0.15	2.13 ± 0.19	1.61 ± 0.18**	2.09 ± 0.17	0.006	0.305	0.009	0.019
**IL-6 (pg/mL) ǂ**	0.19 ± 0.17	0.19 ± 0.18	-0.41 ± 0.18**	0.19 ± 0.18	0.020	0.080	0.038	0.035
**IL-10 (pg/mL) ǂ**	-0.12 ± 0.16	0.49 ± 0.2	0.64 ± 0.16**	0.39 ± 0.19	0.109	0.318	0.012	0.001

Data are presented as model-adjusted least squares means ± SE. ǂ Indicates JN transformed variable means ± SE and P-values. *Mvt-adjusted pairwise P-value ≤ 0.05 for Almond vs. Cookie within the same timepoint. ^Mvt-adjusted pairwise P-value ≤ 0.1 for Almond vs. Cookie within the same timepoint. **Mvt-adjusted pairwise P-value ≤ 0.05 for BL vs. W6 within the same group.

Repeated measures were analyzed using linear mixed-effects models (fixed effects: group, week, group x week; random intercept: participant) with kit plate number included as a covariate for serum markers and derived indices. The Group, Week, and Group x Week columns report Type III tests of fixed effects from these models; pairwise comparisons used multivariate-t (mvt) adjustment. Baseline adjusted Group reports the group effect at Week 6 from a separate baseline-adjusted linear regression. Missing data were handled by maximum likelihood (mixed models) or multiple imputation (regression models).
